# Postoperative Hypophosphatemia as a Prognostic Factor for Postoperative Pancreatic Fistula: A Systematic Review

**DOI:** 10.3390/medicina59020274

**Published:** 2023-01-31

**Authors:** Ieva Grikyte, Povilas Ignatavicius

**Affiliations:** 1 Faculty of Medicine, Medical Academy, Lithuanian University of Health Sciences, 50161 Kaunas, Lithuania; 2 Department of Surgery, Medical Academy, Lithuanian University of Health Sciences, 50161 Kaunas, Lithuania

**Keywords:** postoperative pancreatic fistula, pancreatic leak, pancreatectomy, hypophosphatemia, prediction

## Abstract

*Background and Objectives*: Postoperative pancreatic fistula (POPF) is one of the most challenging complications after pancreatic resections, associated with prolonged hospital stay and high mortality. Early identification of pancreatic fistula is necessary for the treatment to be effective. Several prognostic factors have been identified, although it is unclear which one is the most crucial. Some studies show that post-pancreatectomy hypophosphatemia may be associated with the development of POPF. The aim of this systematic review was to determine whether postoperative hypophosphatemia can be used as a prognostic factor for postoperative pancreatic fistula. *Materials and Methods*: The systematic literature review was performed according to Preferred Reporting Items for Systematic Reviews and Meta-Analyses recommendations (PRISMA) and was registered in the International Prospective Register of Systematic Reviews (PROSPERO). The PubMed, ScienceDirect, and Web of Science databases were systematically searched up to the 31st of January 2022 for studies analyzing postoperative hypophosphatemia as a prognostic factor for POPF. Data including study characteristics, patient characteristics, operation type, definitions of postoperative hypophosphatemia and postoperative pancreatic fistula were extracted. *Results*: Initially, 149 articles were retrieved. After screening and final assessment, 3 retrospective studies with 2893 patients were included in this review. An association between postoperative hypophosphatemia and POPF was found in all included studies. Patients undergoing distal pancreatectomy were more likely to develop severe hypophosphatemia compared to patients undergoing proximal pancreatectomy. Serum phosphate levels on postoperative day 4 (POD 4) and postoperative day 5 (POD 5) remained significantly lower in patients who developed leak-related complications showing a slower recovery of hypophosphatemia from postoperative day 3 (POD 3) through postoperative day 7 (POD 7). Moreover, body mass index (BMI) higher than 30 kg/m^2^, soft pancreatic tissue, abnormal white blood cell count on postoperative day 3 (POD 3), and shorter surgery time were associated with leak-related complications (LRC) and lower phosphate levels. *Conclusions*: Early postoperative hypophosphatemia might be used as a prognostic biomarker for early identification of postoperative pancreatic fistula. However, more studies are needed to better identify significant cut-off levels of postoperative hypophosphatemia and development of hypophosphatemia in the postoperative period.

## 1. Introduction

The rate of patients who develop major complications following pancreatectomy remains high despite recent improvements in surgical and postoperative management and reaches up to 30–60% [1]. Among the most common complications defined and graded by the International Study Group of Pancreatic Surgery (ISGPS) are postoperative pancreatic fistula (POPF) (13–64.9%), post-pancreatectomy hemorrhage (PPH) (3–10%), and delayed gastric emptying (DGE) (13.8–40%) [2,3,4]. Regardless of the type of the procedure and numerous efforts to predict the risk, prevent the formation, and alleviate the severity of POPF, it is the most frequent complication and might result in postoperative abdominal hemorrhage, abscess, sepsis, a longer length of hospital stay, and multiple organ failure leading to fatal outcomes [2,3,5,6,7].

In 2016, the International Study Group on Pancreatic Fistula (ISGPF) released an updated definition of pancreatic fistula, which defines it as a pancreas-derived, enzyme-rich fluid containing abnormal communication between the pancreatic ductal epithelium and another epithelial surface [4]. Furthermore, by its severity, it is classified into biochemical leak (grade A) and clinically significant POPF (grades B and C), which are also known as clinically relevant pancreatic fistulas (CR-PF) and require a change of management or re-operation and are potentially fatal [5]. Clinically relevant POPFs are diagnosed when amylase activity is greater than three times the upper normal serum value in the fluid output from an operatively placed drain, greater than 3 weeks in duration. The main approaches for POPF management include conservative treatment, non-operative catheter intervention, and re-laparotomy. The most used method is drainage of intra-abdominal fluid collection by placing a catheter, but this can be insufficient and lead to re-operation [7]. Usually, by the time re-operation is considered, the patient’s status has significantly deteriorated, and the outcomes are poor. It is noted that the mortality rate after surgical intervention for POPF management ranges between 11% and 35.9% [8,9,10].

There are many studies that have attempted to assess risk factors for POPF and to develop new operative techniques or perioperative care strategies to prevent POPF formation. The success of managing a pancreatic fistula depends on its early identification in the postoperative period and diminishing more serious sequelae [6]. Current risk assessment for leak-related complications (LRC) is insufficient and rarely affects management [11]. Several predictive risk factors for POPF are identified, such as age, gender, body mass index (BMI), anastomotic technique, use of transanastomotic stents, gland texture, histology, history of pancreatitis, involvement of portal vein, but most of these are subjective and contradictory [1,12]. Predictive biomarkers have been commonly investigated in both drain fluid and blood, such as serum albumin and serum lipase, preoperative neutrophil-lymphocyte ratio, serum C-reactive protein, and drainage amylase [13,14,15,16,17]. Timely detection of these biomarkers allows an objective assessment of the presence of clinically relevantPOPF and prompts prophylactic strategies and management. However, despite these indicators being discovered and proven, new biomarkers are investigated to make the risk assessment more accurate. 

There have been a variety of published reports of hypophosphatemia in the postoperative period following various types of surgery. Notably, hypophosphatemia is commonly seen in critically ill patients after severe trauma, during sepsis, and in patients at intensive care units (ICU), at a rate of 30–50% [18]. Phosphorus is an essential electrolyte with different functions and a complex metabolism, whose normal level ranges from 2.5 to 4.5 mg/dL (from 0.80 to 1.45 mmol/L) [19]. The main cause of hypophosphatemia is redistribution across cell membranes, which can be caused by high interleukin levels, drugs, metabolic acidosis, respiratory alkalosis, and high levels of serum catecholamines [19]. Because of acute hypophosphatemia, diverse clinical manifestations may be observed [20,21]. In patients following gastrointestinal surgeries, lower serum phosphate levels are associated with poor outcomes and an increased incidence of organ-specific complications (OSC), including intra-abdominal infections, abscesses, and fistulas [22,23]. Contrarily, following hepatectomy, hypophosphatemia is associated with reduced morbidity and mortality, with several mechanisms being described [11]. Studies show that hypophosphatemia following pancreatectomy can be caused by the same mechanism as hypophosphatemia after hepatectomy [11]. However, there is a lack of studies on hypophosphatemia’s effect on postoperative outcomes following pancreatectomy and the exact mechanism in the postoperative period. Therefore, we conducted this systematic review to determine whether postoperative hypophosphatemia is associated with POPF formation.

## 2. Materials and Methods

### 2.1. Study Design

**Figure 1:**
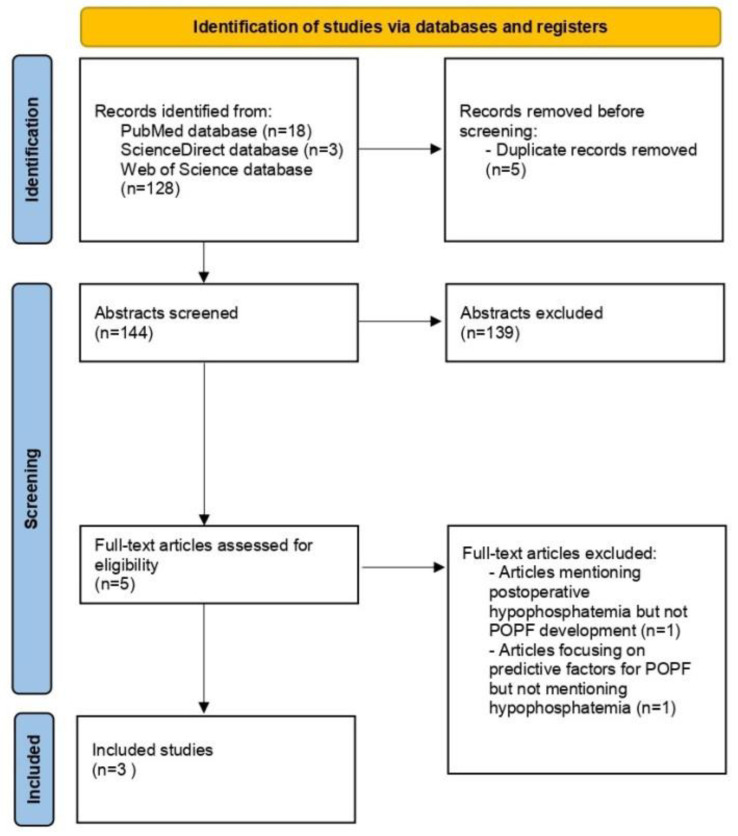
PRISMA flow diagram. One hundred forty-nine articles were identified through database searching. After removal of duplicates, title and abstract screening and further full-text assessment 3 retrospective studies were included in the analysis.

This systematic review was conducted in accordance with the Preferred Reporting Items for Systematic Reviews and Meta-Analyses (PRISMA). The study flow diagram is reported in Figure 1. The study was registered in the International Prospective Register of Systematic Reviews (PROSPERO) system under registration number CRD42022303798 [24].

### 2.2. Search Strategy

The PubMed, ScienceDirect, and Web of Science databases were systematically searched from 31 January 2012 up to 31 January 2022 for studies analyzing postoperative hypophosphatemia as a prognostic factor for postoperative pancreatic fistula. The keywords and search inquiries that were used during the primary stage were as follows: (postoperative hypophosphatemia OR postoperative low phosphorus OR hypophosphatemia OR low phosphorus) AND (pancreatic leakage OR POPF OR postoperative pancreatic fistula OR pancreatic leakage OR pancreatic leak OR anastomotic leakage OR anastomotic insufficiency OR anastomotic failure OR pancreatic fistula OR fistula). The search results from each database were saved in Research Information Systems (RIS) format and imported into Mendeley reference management software. Furthermore, study references from selected articles were also reviewed. No unpublished data were included in this systematic review, and eventually each article was checked against the eligibility criteria.

### 2.3. Selection Criteria

Studies that met the following inclusion criteria were included in this systematic review: (1) studies written in English, (2) retrospective and prospective studies, (3) studies analyzing the association of postoperative hypophosphatemia with the development of postoperative pancreatic fistulas, (4) studies including patients with confirmed hypophosphatemia and postoperative pancreatic fistula following pancreatectomy. The exclusion criteria were as follows: (1) abstracts, case reports, editorials, letters, systematic reviews, and meta-analyses, (2) studies with inadequate or absent data for further analysis, (3) duplicate studies, (4) studies written in a language other than English, (5) studies focusing on preoperative risk factors, (6) studies analyzing postoperative pancreatic fistula following pancreatectomy with no recorded hypophosphatemia.

### 2.4. Data Extraction

Selected studies were evaluated by 2 investigators independently (I.G. and P.I.) and necessary data were extracted: name of the first author and year of publication, study country, study period, study design, sample size, postoperative serum phosphate levels (mg/dL), operation type, the predictive value of hypophosphatemia, pancreatic disease (indication for surgery), the definition of postoperative hypophosphatemia and postoperative pancreatic fistula.

### 2.5. Quality Assessment

Quality assessment of the included studies was performed according to the Newcastle–Ottawa Scale (NOS) for retrospective cohort studies. Studies scoring ≥6 points were considered high quality, with NOS scores ranging from 0 to 9 [25]. The mean value of the 3 included studies was 8.33. Quality assessment was performed by 2 investigators independently (I.G. and P.I.).

## 3. Results

### 3.1. Study Selection 

All retrieved abstracts from the three databases were combined (n = 149) and the duplicates (n = 5) were removed manually before screening (Figure 1). Two reviewers (I.G. and P.I.) independently screened the remaining abstracts (n = 144) and excluded unrelated articles by reading the title and the abstracts (n = 139). Any disagreements were resolved by discussion. The reference lists of original full-text articles and other review articles were further reviewed to search for any missing studies. Subsequently, each article was checked against the eligibility criteria and five articles were selected for the full-text analysis. Two studies were excluded for the following reasons: an article mentioning postoperative hypophosphatemia following pancreatectomy but analyzing complications other than POPF (n = 1) and an article analyzing postoperative complications following pancreatectomy and their predictive risk factors, but not including data on hypophosphatemia (n = 1). Finally, three retrospective cohort studies were included in the systematic review. Extracted data included author details, study details, sample size, postoperative serum phosphate levels (mg/dL), operation type, predictive value, main pancreatic disease, and definitions of postoperative hypophosphatemia and postoperative pancreatic fistula.

### 3.2. Study Characteristics 

Three articles (3) with 2893 patients who underwent pancreatic resection were included in the analysis. Characteristics of each included study are presented in Table 1. The studies spanned from 2018 to 2020. All studies were retrospective analyses [11,22,23]. Patients older than 18 years old who underwent pancreatic resection for any reason were included. The criteria for excluding patients were as follows: patients younger than 18 years old, patients who underwent re-operations or multivisceral, debulking operations. Postoperative serum phosphate levels, incidence of postoperative pancreatic fistulas, demographics, clinicopathological and postoperative observation data, and comorbidities were evaluated [11,22,23]. Various complications such as infectious, abdominal collection/fistula, bleeding/anemia, gastrointestinal, cardiopulmonary, and others were identified. The measurement period for serum phosphate levels varied in the included studies. In the study by Mueller et al., serum phosphate levels were recorded and collected for 5 days postoperatively [11]. The serum phosphate levels were collected at the preoperative visit (within two weeks of surgery) and daily from the day of surgery until discharge in the study by Sadot et al. [22]. Serum phosphate levels were recorded and collected for 10 days postoperatively in the study by Wong et al. [23]. The definition of hypophosphatemia also varied between the included studies. Hypophosphatemia was defined as a serum phosphate level less than 2.5 mg/dL in the study by Wong et al., less than 2.4 mg/dL in the study by Sadot et al., and in the study by Mueller et al., it was not mentioned [11,22,23]. In one of the included studies [21], the authors applied further stratification of hypophosphatemia into different levels of severity: mild (2.5–2.0 mg/dL) and moderate/severe (<2.0 mg/dL). In all studies, POPF was defined according to the International Study Group for Pancreatic Fistulas (ISGPF).

### 3.3. Outcomes

Of the 2893 patients included in the study, the majority (70%) underwent proximal pancreatic resection. For the rest of the patients, distal pancreatectomy (28%) and other pancreatic resections (1.7%) were performed (Table 1). One-third (32%) of patients who underwent pancreatic resection developed fistula-related complications. Among these patients with confirmed POPF, 68.75% had postoperative hypophosphatemia [22,23]. In all three (3) included studies, a clear relation between hypophosphatemia and postoperative pancreatic fistula was shown (Table 2). Patients undergoing distal pancreatectomy were more likely to develop severe hypophosphatemia compared to proximal pancreatectomy patients (29.8% versus 16.0%; *p* < 0.001; *p* = 0.0068 and *p* = 0.0481) [11,23]. Patients with moderate (2.5–2.0 mg/dL) or severe (<2.0 mg/dL) hypophosphatemia were significantly more likely to have fistula-related complications than those who had mild or no hypophosphatemia (56.7% versus 43.3%) [23]. Patients who developed POPF had significantly lower serum phosphate levels on POD 2 and POD 3 (mean 2.2 mg/dL) (Table 2). Following multivariable analysis in the study by Mueller et al., patients who developed POPF had significantly lower serum phosphate levels on POD 0 (3.60 versus 3.75, *p* = 0.01), POD 2 (2.02 versus 2.11, *p* = 0.05), POD 3 (2.12 versus 2.23, *p* = 0.05), POD 4 (2.47 versus 2.60, *p* = 0.009), and POD 5 (2.77 versus 2.94, *p* = 0.003) [11]. In both patient groups, serum phosphate levels started to increase from POD 3. At least one complication was present for patients whose nadir serum phosphate levels were recorded during POD 2 and 3 [11,22,23]. Serum phosphate levels on POD 4 and POD 5 remained significantly lower in patients who developed LRC, showing a slower recovery of hypophosphatemia from POD 3 through POD 7 [11,22]. Moreover, BMI higher than 30 kg/m^2^, soft pancreatic tissue, abnormal white blood cell levels on POD 3, and shorter procedures were associated with LRC and lower phosphate levels [11,22]. Race, the presence or absence of pancreatic cancer, age, gender, and the Charlson comorbidity index were not predictive of POPF [11]. All studies found different significant cut-off levels of serum phosphate in the postoperative period. In the study by Mueller et al., it was 1.75 mg/dL, by Wong et al.—<2.0 mg/dL, by Sadot et al. <2.4 mg/dL [11,22,23].

## 4. Discussion

Our aim was to evaluate postoperative hypophosphatemia as a prognostic factor for POPF in patients undergoing pancreatic resections. After the selection process, three retrospective studies with 2893 patients were included in the systematic review. In all studies, the guidelines for POPF definition according to the International Study Group for Pancreatic Fistulas (ISGPF) were followed. Our review demonstrated that early postoperative hypophosphatemia is potentially an early predictive factor that is reliably associated with POPF formation. In all studies, it was shown that serum phosphate levels started to decrease in patients with POPF from POD 1. All studies had different definitions of hypophosphatemia, and a significant level of hypophosphatemia was also defined differently. In the study by Mueller et al., it was noted that POD 2 phosphate < 1.75 mg/dL predicted an additional 46% increased odds of POPF (OR 1.46 95% CI 1.06–2.01; *p* = 0.02) [11]. In the other two studies, a significant level of serum phosphate was considered as <2.4 mg/dL [22,23]. All studies reported that postoperative hypophosphatemia was associated with the development of POPF and can be used as a prediction factor. However, based on the findings of these studies, no target significant level of serum phosphate level has been set that can be used in prediction, as in each included study a different value was described.

Hypophosphatemia is commonly seen in critically ill patients after severe trauma and burns, in septic patients, and in patients at intensive care units (ICU), at a rate of 30–50% [18]. Phosphate is an essential electrolyte and its lack is associated with increased hospital and ICU length of stay and appears to be a biomarker of disease severity [26]. The development of hypophosphatemia in the body is explained by four main mechanisms: redistribution of phosphate from the extracellular fluids into cells, increased renal excretion, decreased absorption in the gastrointestinal tract, and loss due to renal replacement therapy [27]. Redistribution of phosphate can be caused by high interleukin levels, drugs, metabolic acidosis, respiratory alkalosis, and high levels of serum catecholamines [19]. The definition of the hypophosphatemia-defining threshold varies, but a commonly accepted definition of hypophosphatemia is <2.5 mg/dL (<0.81 mmol/L) [27,28,29]. In this systematic review, all authors described hypophosphatemia differently. In the study by Sadot et al., hypophosphatemia was defined as serum levels less than 2.4 mg/dL, in the study by Wong et al., it was level less than 2.5 mg/dL, and in the study by Mueller et al., it was not specified [11,22,23]. Outcomes of hypophosphatemia include neuromuscular disturbances, encephalopathy, paresthesia, ileus, and respiratory failure [20,30,31]. Postoperatively, hypophosphatemia is associated with more severe complications, poor outcomes, and an increased incidence of organ-specific complications (OSC), including intra-abdominal infections, abscesses, and fistulas [22,23]. Contrarily, following hepatectomy, hypophosphatemia is associated with reduced morbidity and mortality, with several mechanisms being described [11]. However, there is a lack of studies on hypophosphatemia’s effect on postoperative outcomes following pancreatectomy and the exact mechanism in the postoperative period.

Several case reports and small case series have reported the frequent occurrence of hypophosphatemia after liver resections. A study by Hallet et al. reported that postoperative hypophosphatemia after liver resection may not only represent a recovery of initial liver insufficiency (ILI) but may also reduce the risk of surgical complications associated with ILI [32]. In the study by Squires et al., patients who underwent a major hepatectomy were identified and postoperative serum phosphorus levels were assessed. It was noted that elevated phosphorus levels > 2.4 mg/dL at POD 2 and a delayed nadir in phosphorus beyond POD 3 are associated with increased hepatic insufficiency, major complications, and early mortality. It has also been pointed out that insufficient liver remnant regeneration may be indicated by a failure to develop hypophosphatemia within 72 h after a major hepatectomy [33]. Post-hepatectomy hypophosphatemia has been explained by a variety of hypotheses, but its exact mechanism remains unknown. One of the hypotheses explains it by increased flux of serum phosphate into hepatocytes needed for the high energy-consuming liver regeneration process [34]. This hypothesis has been analyzed in several studies that suggest that hypophosphatemia correlates with liver regeneration [35,36]. It has been found that the absence of hypophosphatemia or an early nadir after partial hepatectomy increases 30-day mortality rates after partial hepatectomy [33,37]. Salem et al. introduced another hypothesis, which claims that the predominant cause of hypophosphatemia is transient isolated hyperphosphaturia rather than an increase in phosphate utilization [38]. In a recent study, it was found, that serum nicotinamide phosphoribosyl-transferase (NAMPT) is a phosphaturic factor associated with phosphaturia, following pancreatectomy and hepatectomy [39]. However, despite the similar pathophysiological mechanisms, postoperative hypophosphatemia following pancreatectomy is associated with a higher rate of complications [22,39].

Postoperative pancreatic fistulas result in complex and prolonged inpatient care and a significant cost burden. An estimated 13% to 60% of patients suffer from a postoperative pancreatic fistula following pancreatic resection, and it is the main cause of major morbidity and mortality [1,40,41]. The incidence of POPF after distal pancreatectomy has been reported to be between 18.6 and 64.9%, while the incidence of clinically relevant fistula after proximal pancreatectomy has been reported to range from 13% to 36% [20,31,42,43]. An early prediction of a pancreatic fistula is important since it identifies those who need to be closely monitored and may require alterations to the further treatment plan. By detecting POPF early, it is possible to prevent the development of potentially lethal consequences. Hemorrhage and sepsis are the two most frequent sequelae of POPF. The consequences of these complications may include prolonged hospital stay, delayed gastric emptying, enteric fistulae, multiorgan failure, and death [44]. An update on pancreatic fistula diagnosis and grading was published by the International Study Group on Pancreatic Surgery (ISGPS) in 2016. Pancreatic fistulas were classified into biochemical leak (grade A) and clinically relevant POPFs (grades B and C) [5]. A biochemical leak is characterized by elevated drain amylase levels, does not lead to any clinical complications, and can be managed conservatively. There is evidence that the more severe the fistula grade, the more severe the clinical consequences and the greater the cost of hospitalization [6]. Moreover, late detection delays treatment and poses a meaningful threat to perioperative survival [45]. POPF has been studied through many cohort studies, identifying risk factors that increase the likelihood of developing it. The management of POPF includes its early recognition and prevention of further life-threatening sequelae. Several factors have been consistently shown to predict POPF after pancreatoduodenectomy (PD), including soft gland texture, non-pancreatic cancer pathology, small pancreatic duct diameter (<3 mm), and high intraoperative blood loss (>1000 mL) [10]. In the prediction of POPF, preoperative, intraoperative, and postoperative risk factors are distinguished. During the postoperative period, macroscopic and biochemical analyses of drain fluid are significant predictors of POPF development. Macroscopically, in the first few postoperative days, a red-brown drain fluid can be observed, which is thought to be caused by the enzymatic breakdown of proteins, which results from leakage of protease-rich pancreatic fluid [4]. The most evaluated biochemical parameter in the postoperative period is the drainage amylase concentration [46]. Other biochemical factors include urinary trypsinogen-2, serum amylase, and serum lipase measurements [17,47]. Recently published studies have been focusing on discovering new biochemical predictors, which would let physicians detect POPF early. A study by Lale et al. demonstrated that decreased mean platelet count ratio (MPR) is a strong predictor for clinically relevant POPFs and should be considered when deciding treatment strategies [48]. Following pancreaticoduodenectomy (PD), higher arterial lactate (LCT) levels were also identified as a predictive marker for POPF in a study by Sakamoto et al., which is explained by decreased pancreatic blood flow after PD [48]. Early identification is the main key in the management of POPF, which makes the understanding of predictive risk factors and the underlying pathophysiological mechanisms crucial. Identification of electrolyte abnormalities and changes in serum biochemical parameters may be useful indicators of complications, allowing physicians to have sufficient time for an intervention.

Although hypophosphatemia is associated with opposite outcomes from hepatectomy when following pancreatectomy, the proposed mechanism is similar [39]. Following liver resections, the incidence of morbidity and mortality has been decreased in patients with hypophosphatemia [32,33]. Moreover, several proinflammatory cytokines and enzymes have been correlated with hypophosphatemia [39,49,50]. It was found that patients who underwent pancreatectomy displayed significantly higher levels of hyperphosphaturia than those who underwent hepatectomy [39]. In addition, it was noted that NAMPT may increase postoperatively due to the inflammatory changes and increased cellular metabolism, independent of the type of resection performed. In a significant proportion of patients who underwent pancreatic resection, inflammation has been found to play a critical role in the development of POPF [51]. It has been shown that inflammatory response reduces phosphate levels due to an increased metabolic state, increased cytokine release, inflammatory cell activation, and transcellular shifts, among other causes [29,50,52]. Consequently, postoperative acute pancreatitis and hyperphosphaturia may have a cyclical relationship, which leads to the development of hypophosphatemia and POPF. A BMI higher than 30 kg/m^2^, soft pancreatic tissue, abnormal white blood cell levels, and shorter procedures were associated with LRC and lower phosphate levels [11,22]. The pathophysiological mechanism of hypophosphatemia in relation to fistula formation is still unclear, as phosphate metabolism can be influenced by several factors after pancreatic surgery. However, the higher frequency of hypophosphatemia in patients after pancreatectomy supports the notion that moderate/severe hypophosphatemia is associated with fistula-related complications [11,22,23].

This systematic review is the first review of its kind to be published on this subject. However, there are several limitations. Only three studies were included in this systematic review and all studies were retrospective, increasing the chances of publication bias. Due to the lack of data on this topic, the results should be interpreted with caution. Secondly, high heterogeneity among the studies was observed. Serum phosphate cutoff values differed in the included studies, further contributing to increased heterogeneity. Based on this review, it can be argued that one of the potential postoperative follow-up indicators for patients undergoing pancreatic resection surgery might be serum phosphate levels. 

## 5. Conclusions

This review shows that early postoperative hypophosphatemia may allow early identification of patients at risk for developing pancreatic fistula. The observation of severe hypophosphatemia on POD 2 and POD 3 might be a good biomarker for predicting POPF. However, more studies are needed to clarify the pathophysiological association of hypophosphatemia with the development of pancreatic fistula. In addition, it is important to identify significant cut-off levels of postoperative hypophosphatemia and changes in phosphorus levels during the postoperative period for better stratification of patients.

## Figures and Tables

**Table 1:** Description of the studies included in the systematic review.

First Author, Year	Study Country	Study Period	Study Design	NOS Score	Operation Type			Definition of Hypophosphatemia (mg/dL)
					Proximal Pancreatectomy, n (%)	Distal Pancreatectomy, n (%)	Other, n (%) *	
P. Wong, 2020 [23]	USA	2009–2017	Retrospective cohort	9	173 (61.13%)	92 (32.5%)	18 (6.36%)	<2.5
J. Mueller, 2018 [11]	USA	2001–2017	Retrospective cohort	9	1587 (68.67%)	724 (31.33%)	31 (1.3%)	<1.75
E. Sadot, 2018 [22]	USA	2011–2012	Retrospective cohort	8	268 (100%)			<2.4

* Other pancreatic operations included: enucleation, total pancreatectomy, other unnamed operations.

**Table 2:** Comparison of patients’ serum phosphate levels (mean) according to whether they developed POPF or not (mg/dL).

Postoperative Day	P. Wong, 2020 [23]		J. Mueller, 2018 [11]			E. Sadot, 2018 [22]
	Fistula-Related Complications	No Complications	Fistula-Related Complications	No Complications	Fistula-Related Complications	No Complications
POD0	4.12	4.03	3.60 *	3.75 *	3.72	3.72
POD1	3.65	3.58	3.04	3.10	3.10	3.10
POD2	2.35 *	2.28 *	2.02 *	2.11 *	1.89	1.89
POD3	2.15	2.35	2.12	2.23	2.01 *	2.20 *
POD4	2.53	2.77	2.47 *	2.60 *	2.38 *	2.69 *
POD5	2.86 *	3.18 *	2.77 *	2.94 *	2.84 *	3.19 *

* Pairs with statistically significant (*p* < 0.01) difference.

## Data Availability

Not applicable.
